# Understanding perception and acceptability of a gonorrhoea vaccine among young migrants in England for effective scale-up

**DOI:** 10.1177/26334941261448067

**Published:** 2026-05-07

**Authors:** Obasanjo Bolarinwa

**Affiliations:** Department of Global Healthcare Management, York St John University, 1 Clove Street, East India, London E14 2BA, UK

**Keywords:** England, gonorrhoea vaccine, sexual health services, vaccine acceptability, young migrants

## Background

England has begun a world-first gonorrhoea vaccination programme using the serogroup B meningococcal vaccine, also known as 4CMenB, in specialist sexual health services for those at the highest risk, as announced on 4 August 2025.^1^ This shift towards prevention comes amid concerns about antimicrobial resistance and recent peaks in gonorrhoea diagnoses.^2^ Evidence indicates that 4CMenB confers partial cross-protection against Neisseria gonorrhoeae, though effectiveness is moderate and may wane over time, which places a premium on programme design, targeting and uptake.^3^ To scale up subsequent deployment to additional priority groups, there is a clear need to understand how young migrants in the United Kingdom perceive this vaccine and whether they would accept it.

Young migrants face distinct social and structural barriers to sexual and reproductive health services in high-income settings, including language, precarious work and housing, fear of immigration surveillance, limited system navigation and stigma.^4,5^ These barriers reduce service engagement and can depress vaccine uptake. During the COVID-19 response, ethnic inequalities in vaccine confidence and uptake among UK populations were well documented, underlining the importance of trust, culturally responsive communication and engagement with trusted messengers. Without dedicated formative research, a gonorrhoea vaccine offer may reproduce these inequities, limiting both direct protection and population-level impact.^6^

Inquiry should therefore examine perceptions, beliefs and practical considerations specific to young migrants. First, clarity is needed on awareness of gonorrhoea risks and complications, the difference between a meningitis B vaccine and its off-label or cross-protective use for gonorrhoea and how partial effectiveness is interpreted in decisions to vaccinate. Second, research should identify preferred access points and delivery models, including confidentiality expectations in both sexual health clinics and mobile or community settings. This should also include multilingual materials and the role of peers or community health champions. Third, it should explore concerns about data sharing, costs and entitlements, and how these interact with immigration status. Insights from this work should inform communication, consent processes, eligibility messaging and co-designed outreach with community partners directly.

Acceptability research should combine qualitative methods, such as focus groups and in-depth interviews, with quantitative stated-preference approaches that can estimate trade-offs between attributes that matter to young migrants, for example, the number of doses, expected protection, side effects, place of delivery, waiting times, reminder systems and perceived privacy. Findings can inform segmented communication and service design that reflect the diversity within migrant populations, taking into account factors such as age, gender, country of origin, migration route, language and sexual orientation. Where appropriate, terminology should be community-led and sensitive to ongoing debates in the UK about collective labels while remaining operationally precise for surveillance and programme delivery.

Finally, understanding acceptability before scale-up is a matter of programme efficiency and equity. The 4CMenB offer provides only moderate protection and will deliver the greatest benefit if uptake is high in those at greatest risk. With resistant gonorrhoea rising and services under pressure, co-produced strategies that reduce structural barriers and build trust are essential to realise vaccine impact for young migrants and to avoid widening disparities in sexual health outcomes.

## Declarations

**Ethics approval and consent to participate:** Not applicable.

**Consent for publication:** Not applicable.

**Availability of data and materials:** Not applicable.
